# Prevalence of Malocclusion in Jaipur, India

**DOI:** 10.5005/jp-journals-10005-1036

**Published:** 2009-04-26

**Authors:** Mridula Trehan, Vinay K Chugh, Sunil Sharma

**Affiliations:** 1 Professor and Head, Department of Orthodontics and Dentofacial Orthopedics, Mahatma Gandhi Dental College and Hospital Jaipur, India; 2 Senior Lecturer, Department of Orthodontics and Dentofacial Orthopedics, Mahatma Gandhi Dental College and Hospital Jaipur, India; 3 Vice Principal, Mahatma Gandhi Dental College and Hospital, Jaipur, India

**Keywords:** Malocclusion, Angle’s classification, prevalence.

## Abstract

A study was undertaken to determine the prevalence of malocclusion in Jaipur city, India. A total of 700 subjects, in the age group of 16-26 years were divided into five groups of normal occlusion, Angle’s Class I, Class II Div 1, Class II Div 2 and Class III malocclusion. The results revealed that the prevalence of malocclusion was 66.3%, with the majority of them having Class I malocclusion (57.9%), while the prevalence of Class III malocclusion was found to be the least (1.4%). There was no statistically significant gender difference among the subjects studied.

## INTRODUCTION

Normal alignment of teeth not only contributes to the oral health but also goes a long way in the overall well-being and personality of an individual. Correct tooth position is an important factor for esthetics, function and for overall preservation or restoration of dental health. While dental caries has been regarded as the one of the major dental disease throughout the world, malocclusion is a close runnerup. The morphogenetic nature of most malocclusions assures us that this dentofacial problem will continue to demand the best that dentistry can offer for a long time, indeed. Many organized population surveys have been carried out in different parts of the world with the objective of estimating prevalence of malocclusion and orthodontic treatment needs.

The prevalence of malocclusion varies greatly in different parts of the world, in different ethnic groups and people of different origins. The prevalence of malocclusion among Indian population has been reported to be as low as 19.6% (Miglani DC et al in 1965) and as high as 90% in Delhi by Sidhu.^4^ To assign a treatment plan and to work out on the treatment needs of a particular group or population, it is mandatory to know the trends of occurrence of various malocclusions. As there is a lack of statistical data on malocclusions in this particular geographical area, a study was conducted on 700 patients in the city of Jaipur, the capital of Rajasthan, to identify the distribution of malocclusion. Though there is no single way to classify malocclusion, the most commonly and universally accepted Angle’s classification was used, due to its simplicity.

## MATERIAL AND METHOD

The present study was carried out on 700 subjects ranging in ages from 16 to 26 years. The subjects were selected randomly from the patients reporting to the OPD of Mahatma Gandhi Dental College and Hospital, Jaipur. The criteria for selection of the subjects were as follows:

1. All permanent teeth present in each arch (excluding third molars) and in a sufficient state of eruption.
2. No previous history of orthodontic treatment in either arch.
3. No large coronal restoration that might have altered both coronal shape and size.

**Table 1:** Table 1: Distribution of sample

	Group I		Normal occlusion
	Group II		Class I malocclusion
	Group III		Class II Div 1 malocclusion
	Group IV		Class II Div 2 malocclusion
	Group V		Class III malocclusion

All occlusal relationships were evaluated at a centric occlusion position which was achieved by asking the subject to swallow and then to bite on his or her teeth. The sample was divided into normal occlusion group and malocclusion groups, on the basis of Angle’s classification (Table 1).

**Group I:*** Normal occlusion (NO):* Only those subjects were included in the study which on clinical evaluation showed bilateral Angle’s Class I molar relationship with acceptable overjet and overbite and well-aligned arches.

**Group II:** Showed bilateral Angle’s class I molar relationship with one or more of these characteristics: crowded incisors (Dewey type 1), protruded maxillary incisors (Dewey type 2), anterior cross-bite (Dewey type 3), unilateral or bilateral posterior cross-bite (Dewey type 4,) mesial drift of molars (Dewey type 5), anterior or posterior open bite and deep anterior overbite.

**Group III:** Class II Div 1 malocclusion

**Group IV:** Class II Div 2 malocclusion

**Group V:** Class III malocclusion

The collected data were tabulated and analyzed statistically.

## RESULTS

The occlusal classification of the subjects is shown in Table 2.

**Fig. 1.**
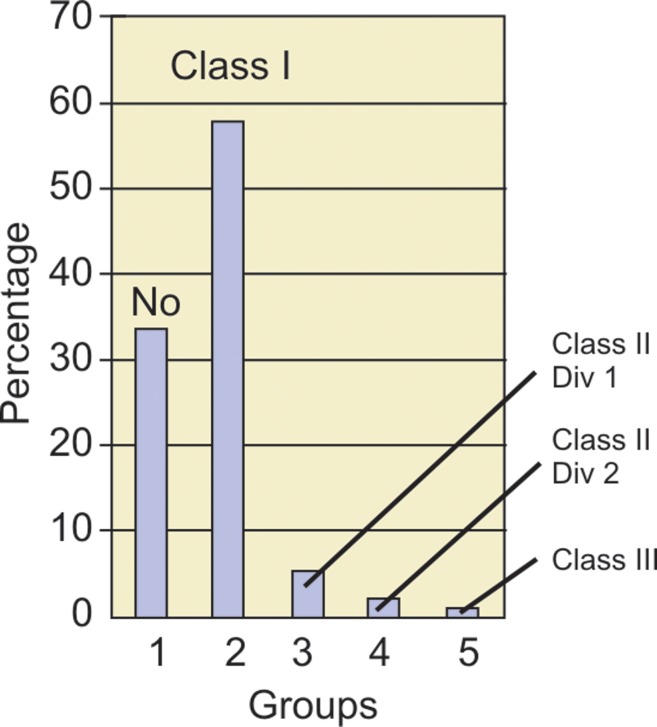
Percentage distribution of groups

Table 2 (Fig. 1) shows that normal occlusion was found in 33.3% of the subjects and 66.7% of the subjects had malocclusion. Prevalence of Angle’s class I malocclusion was the highest (57.9%) and that of Angle’s class III malocclusion was the least (1.4%).

Table 3 shows the gender distribution of normal occlusion and various types of malocclusion. 32.8% of the males had normal occlusion while Class II Div 2 malocclusion was least prevalent (1.1%). 35.5% of the females were found to have normal occlusion while prevalence of class III malocclusion was least.

**Table 2:** Table 2: Occlusal classification

*Occlusion*		*N*		*%*
Normal occlusion (NO)		223		33.3
Angle’s class I malocclusion		405		57.9
Angle’s class II Div 1 malocclusion		39		5.5
Angle’s class II Div 2 malocclusion		13		1.9
Angle’s class III malocclusion		10		1.4
Total		700		100.0

**Table 3:** Table 3: Gender distribution of occlusal variations

*Occlusal classfication*					*Male*							*Female*				
					*N*			*%*				*N*			*%*	
Normal occlusion					184			32.8				49			35.5	
Class I					334			59.6				71			51.5	
Class I Div 1					28			5.0				11			8.0	
Class II Div 2					6			1.1				7			5.0	
Class III					10			1.5				0			0	

## DISCUSSION

The study showed that 66.3% of the subjects surveyed had malocclusion. This is somewhat similar to the findings of Das et al,^2^ who conducted an epidemiological study of malocclusion in the age group of 8-12 years in Bangalore city in 2008, and reported a high incidence of malocclusion of 71%. The findings of the present study are in disagreement with those of Kharbanda et al^3^ who have found 36.6% prevalence of malocclusion in Delhi, while Sidhu^4^ in his study found higher prevalence rate of Angle’s malocclusion of 90% in the age group of 6-30 years.

Angle’s class I malocclusion (57.9%) was more common than Angle’s class II div 1 malocclusion (5.5%). This is similar to the findings of Das et al^2^ who reported 62% class I malocclusion and 7% class II Div 1 malocclusion. The prevalence of class II Div 2 and Class III malocclusion were low, i.e. 1.9% and 1.4% respectively. This is similar to the findings of Singh et al,^3^ who reported prevalence of class II Div 2 malocclusion of 5.85% and prevalence of class III malocclusion of 3.17% in his study of distribution of malocclusion among North Indians seeking orthodontic treatment.

There was no statistically significant difference between males and females either in the prevalence of malocclusion.

## CONCLUSION

From this study, the following conclusions have been drawn:

1. Prevalence of malocclusion was found to be 66.7%.
2. Angle’s class I malocclusion was more prevalent as compared to the other types of malocclusion.
3. There was no statistically significant gender difference among the subjects studied.

The prevalence of malocclusion is high, a reason to continue training professionals to care for those patients in need of treatment. The functional, esthetic, and psychologic benefits of orthodontic treatment ensure a continued seeking out of these services by those afflicted with malocclusion.
